# Hydrolysate from Mussel *Mytilus galloprovincialis* Meat: Enzymatic Hydrolysis, Optimization and Bioactive Properties

**DOI:** 10.3390/molecules26175228

**Published:** 2021-08-28

**Authors:** Sara A. Cunha, Rita de Castro, Ezequiel R. Coscueta, Manuela Pintado

**Affiliations:** CBQF—Centro de Biotecnologia e Química Fina—Laboratório Associado, Escola Superior de Biotecnologia, Universidade Católica Portuguesa Rua Diogo Botelho 1327, 4169-005 Porto, Portugal; scunha@ucp.pt (S.A.C.); rita_de_castro@live.com.pt (R.d.C.); ecoscueta@ucp.pt (E.R.C.)

**Keywords:** antioxidant, anti-hypertensive, proteins, sustainability, marine species, marine hydrolysates

## Abstract

Mussel production generates losses and waste since their commercialisation must be aligned with target market criteria. Since mussels are rich in proteins, their meat can be explored as a source of bioactive hydrolysates. Thus, the main objective of this study was to establish the optimal production conditions through two Box–Behnken designs to produce, by enzymatic hydrolysis (using subtilisin and corolase), hydrolysates rich in proteins and with bioactive properties. The factorial design allowed for the evaluation of the effects of three factors (hydrolysis temperature, enzyme ratio, and hydrolysis time) on protein/peptides release as well as antioxidant and anti-hypertensive properties of the hydrolysates. The hydrolysates produced using the optimised conditions using the subtilisin protease showed 45.0 ± 0.38% of protein, antioxidant activity via ORAC method of 485.63 ± 60.65 µmol TE/g of hydrolysate, and an IC_50_ for the inhibition of ACE of 1.0 ± 0.56 mg of protein/mL. The hydrolysates produced using corolase showed 46.35 ± 1.12% of protein, antioxidant activity of 389.48 ± 0.21 µmol TE/g of hydrolysate, and an IC_50_ for the inhibition of ACE of 3.7 ± 0.33 mg of protein/mL. Mussel meat losses and waste can be used as a source of hydrolysates rich in peptides with relevant bioactive properties, and showing potential for use as ingredients in different industries, such as food and cosmetics, contributing to a circular economy and reducing world waste.

## 1. Introduction

Mussels are highly consumed in several countries. Asia and Europe are considered the leading producers, estimated to produce about 1.05 and 0.5 million tonnes of mussel per year, respectively [1,2]. Mussel consumption has several advantages, for both the environment and consumers. Environmentally, mussel farming can be done with minimal greenhouse gas emissions, and thus low carbon footprint and few environmental impacts [3]. Mussels farming produces about 0.6 kg of CO_2_ emission/kg edible product, while beef produces about 19.0–36.7 kg of CO_2_ emission/kg edible product [3]. For consumers, mussel meat has low fat and low calories. Still, more importantly, mussels are a rich source of sodium, selenium, vitamin B twelve, zinc [1], and an interesting source of proteins since they are composed of about 58.7% of protein on a dry weight basis [4]. Due to their protein-rich meat, mussels have been described as a source of bioactive peptides with relevant biological properties. Bioactive peptides are fragments that are inert when inside proteins but show different properties when broken from the original protein [5]. Thus, enzymatic hydrolysis with proteases seems an interesting approach for obtaining bioactive extracts since these enzymes may break mussel proteins into smaller peptides, which may be associated with other biological and functional properties [6]. Different enzymes have been used to produce bioactive peptides from mussels, such as pepsin [7], flavourzyme [8], papain [8], and trypsin [9]. Marine species have often been described as a source of bioactive peptides, and some bioactivities have been associated to mussel peptides, such as antioxidant [10], anti-hypertensive [11], antimicrobial [12], anticancer [7], anti-inflammatory [13], anticoagulant [14], antidiabetic [15], and antiviral [16]. As far as we know, *Mytilus galloprovincialis* bioactive extracts are not so extensively explored, with the main studies being developed with *Mytilus coruscus* and *Mytilus edulis*. The farming of *M. galloprovincialis*, also known as the Mediterranean mussel, has mainly been developing along the Spanish Atlantic coast and in the Mediterranean area [2].

Mussel commercialisation generates losses and waste since they are submitted to a pre-selection before being delivered for sale, resulting in the rejection of broken mussels or those which fail to meet established criteria in the target market [17,18,19,20]. It is estimated that about 27% of produced mussels are discarded [4,20]. Thus, mussel meat waste can be used to produce bioactive hydrolysates with interesting properties for food, cosmetic, pharmaceutical, and nutraceutical industrial applications.

In this work, *Mytilus galloprovincialis* meat was submitted to different conditions according to two factorial designs to produce hydrolysates with a maximum level of soluble rich proteins/peptides and bioactive properties, more specifically antioxidant and anti-hypertensive. The hydrolysates were produced by enzymatic hydrolysis using two different enzymes, subtilisin and corolase. Therefore, this study aims to valorise mussel meat by creating bioactive hydrolysates with potential for various industries.

## 2. Results

### 2.1. Mytilus Galloprovincialis Characterisation

The mussel’s meat was received under refrigeration on the day of capture. It was characterised before being minced according to a few nutritional characteristics, showing a protein content of 70.50 ± 13.44%, 90.30 ± 4.24% moisture, and 5.00 ± 0.00% lipids.

### 2.2. Optimisation of the Production of Hydrolysates Rich in Proteins and Bioactive Properties

Enzymatic hydrolysis is one of the main used methods to produce bioactive extracts, and is described for the mussel species *M. coruscus*, *M. edulis*, and *M. galloprovincialis*. Several enzymes have been used in the mussel species, such as papain [8], flavourzyme [21], and the digestive enzymes pepsin [7] and trypsin [9]. In this work, two different proteases, subtilisin and corolase, were used to produce hydrolysates rich in proteins/peptides and with antioxidant and anti-hypertensive properties. To understand the conditions that allow to achieve the production of hydrolysates with a better protein% and higher bioactive properties, two experimental designs were made, one for each protease. Different combinations of factors in an enzymatic hydrolysis may lead to differing effectiveness. Thus, we have used a factorial design with 15 combinations of the enzyme (%), temperature (°C), and hydrolysis time (h) for each protease.

Mussel meat was initially minced until homogenised, thus creating uniformed biomass used for all the 60 hydrolysis reactions performed. Then, all the hydrolysis were performed using ultrapure water as the solvent, at 7.5 pH, with a ratio of 1:2 (*w*:*v*) (mussel biomass:water). The 60 hydrolysis reactions were performed using the factors combinations matrix generated by the experimental design, and protein/peptides and bioactivities were measured in the resulting supernatants.

In an enzymatic reaction, different factors combinations may lead to the production of extracts with different characteristics. Thus, the factorial designs allowed us to understand the best combination for optimising protein/peptide bioactive extract production. For both designs, the matrix and obtained results are presented as well as the Pareto charts obtained for each evaluated response, indicating the factors with the greatest influence for each variable studied. For each evaluated response, a multiple regression analysis of the experimental data allowed to obtain a model that can predict the responses and these are shown as Equations (1)–(6). An analysis of variance (ANOVA) was performed to evaluate the significance of each effect and to determine the factors that significantly affected protein % as well as antioxidant and anti-hypertensive properties.

#### 2.2.1. Experimental Design with Subtilisin Protease

**Figure 1:**
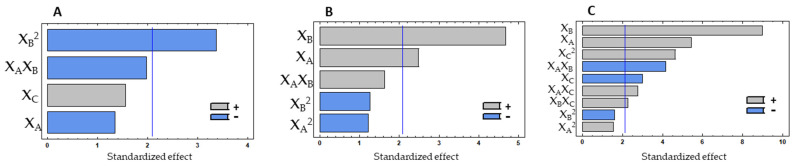
Pareto charts with the effect of three experimental factors, in decreasing order, obtained for protein % (**A**), antioxidant (**B**) and anti-hypertensive (**C**) properties in the experimental design with subtilisin, showing the most influent factors. The vertical lines in the pareto charts represent the level of significance (*p* = 0.05). X_A_—Enzyme %; X_B_—Hydrolysis temperature; X_C_—Hydrolysis time.

**Figure 2:**
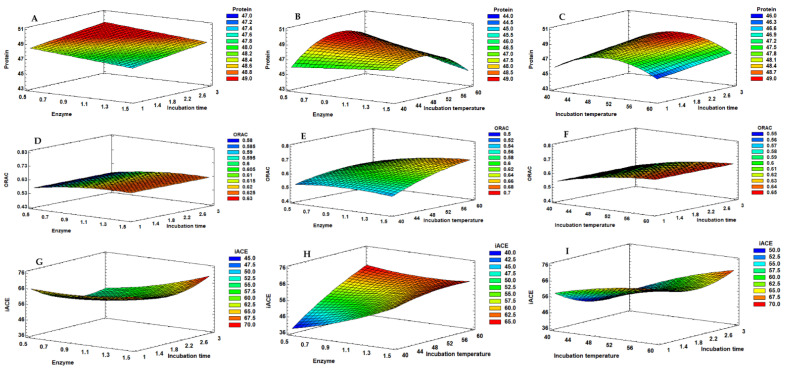
Response surface graphs corresponding to the combined effect of enzyme % (X_A_), hydrolysis temperature (X_B_) and hydrolysis time (X_C_), on the protein (**A**–**C**), ORAC (**D**–**F**) and iACE (**G**–**I**) responses obtained for the mussel extracts produced with subtilisin.

Table 1 shows the experimental design matrix and the results obtained for the Box–Behnken factorial design performed with the subtilisin protease. Figure 1 shows the Pareto charts obtained for the design performed with the subtilisin protease. Figure 2 illustrates the response surface graphics obtained for the independent variables tested, showing their interactions when studying each dependent variable.

Protein content did not show significant variations among all the combinations tested since the range of variation for results was 45.05–49.12% of protein in the final hydrolysate. The Pareto chart (Figure 1A) and the ANOVA analysis (Table 2) show that protein release was not influenced by the linear factors, but only by the quadratic effect of the temperature. The temperature quadratic coefficient showed a negative effect, indicating an increase in protein % at intermediate values. Since none of the factors had a significant effect, the model was adjusted to best fit. Thus, only the linear effect of enzyme% and time, as well as the linear interaction between enzyme% and temperature and the quadratic effect of temperature were considered (Table 2). By multiple regression, the predicted response for the protein % could be expressed by the model in Equation (1).

Protein % = −13.1089 + 10.2225 × X_A_ + 0.635 × X_C_ − 0.22625 × X_A_X_B_ − 0.0200714 × X_B_ ^2^ (1)

The final adjusted model showed a significant fit (*p* > 0.050). However, the R^2^ = 38.52%, indicating that only 38.52% of the variability observed may be explained by the model. This indicates that, for obtaining hydrolysates rich in proteins/peptides, the minimum studied variables could be used (0.5% enzyme, 40 °C, 1 h) to achieve protein release superior to 40%. Although, since this work aims to produce hydrolysates with bioactive properties, the antioxidant and anti-hypertensive properties must be analysed.

The antioxidant property, measured in the soluble hydrolysates by ORAC, was only significantly influenced by the linear effect of the hydrolysis temperature (X_B_) and enzyme % (X_A_) (*p* < 0.050), with hydrolysis time (X_C_) not showing a significant effect on this property (Figure 1B). Temperature and enzyme % positively affected antioxidant property, meaning that the response is directly proportional to the tested values. For ORAC, the interactions between variables did not show a significant effect, the same being verified for the quadratic effect of the three studied variables. By multiple regression, the predicted response for the ORAC could be obtained by the model in Equation (2).

ORAC = −0.0333894 − 0.0436106 × X_A_ + 0.0206284 × X_B_ (2)

Analysing the ANOVA results (Table 3), the final adjusted model showed a significant fit (*p* > 0.050). However, the R^2^ = 45.65%, indicating that only about 45.65% of the variability in the antioxidant activity may be explained by the model.

The ACE inhibition % was evaluated using a concentration of 10 mg hydrolysate/mL. The linear effects of the three studied factors (X_A_, X_B_ and X_C_) were significant for the variations observed on the ACE inhibition %, as well as the linear interaction between all the variables (X_A_X_B_, X_A_X_C_, and X_B_X_C_) and the quadratic effect of the hydrolysis time (X_C_^2^) (Figure 1C). Thus, seven effects showed a *p* < 0.050, while the other two effects (X_A_^2^ and X_B_^2^) were not significant and consequently removed from the model. Temperature and enzyme % showed a high impact on the response. The linear effect of hydrolysis time (X_C_) and the linear interaction between temperature and enzyme % showed a negative contribution, which means that there is an increase of the ACE inhibition at intermediate values. Thus, the longer the reaction, the lower the inhibition of ACE will be, which can mean that the higher extension of the hydrolysis leads to the formation of peptides with less activity against ACE. The quadratic effect of hydrolysis time (X_C_^2^) showed a positive effect on the response, which means that, considering the negative effect verified for X_C_, higher values of iACE were achieved near the minimum values studied. The other linear factors with a significant contribution positively affected the results, meaning that the anti-hypertensive potential is enhanced by the increase in enzyme % and by the higher temperatures.

By multiple regression, the predicted response for the iACE could be obtained by the model in Equation (3).

iACE = −24.3485 + 38.3047 × X_A_ + 3.41606 × X_B_ − 45.3303 × X_C_ − 1.13715 × X_A_ × X_B_ + 6.3675 × X_A_ × X_C_ − 0.0200471 × X_B_^2^ + 0.2635 × X_B_ × X_C_ + 5.83804 × X_C_^2^ (3)

Analysing the ANOVA results (Table 4), the final adjusted model showed a significant fit (*p* > 0.050) and an R^2^ = 81.30%, indicating the variability observed in terms of ACE inhibition is highly explained by the model.

After analysing the results for each variable, we intended to maximise the hydrolysis in order to achieve higher protein content and antioxidant and anti-hypertensive properties. For that, a Derringer’s desirability analysis was performed [22] (Table 5). The optimum conditions predicted were 52.5 °C, 1.5% of subtilisin, and an hydrolysis time of 3 h (Table 5).

The experiments were validated in triplicate, using the same biomass quantities and solvent volumes but adapting temperature to 52 °C, to work practically. The obtained protein content, ORAC, and ACE inhibition values were 46.70 ± 0.36%, 0.54 ± 0.029 µmol TE/mg hydrolysate and 70.21 ± 2.9%, respectively (Table 6). When comparing the results predicted by the factorial design, the obtained results were similar to those predicted by the design.

After optimising the enzymatic hydrolysis reaction, a scale-up was performed, in triplicate, increasing the amount of mussel biomass and solvent by 15 times and maintaining the tested ratio. The temperature was adjusted to 50 °C, to be easily adapted to an industrial scale. The obtained protein content, ORAC, and IC_50_ for ACE inhibition values were 45.0 ± 0.38, 0.49 ± 0.061 µmol TE/mg hydrolysate, and 1.0 ± 0.56 mg protein/mL, respectively (Table 6). The obtained results indicated that the increase in the proportions seems to influence the evaluated responses negatively. Nevertheless, the obtained hydrolysates showed interesting protein/peptide values and antioxidant potential, making these extracts an interesting protein source and a potential ingredient for functional food or cosmetic formulations focused on anti-ageing properties.

#### 2.2.2. Experimental Design with the Corolase Protease

**Figure 3:**
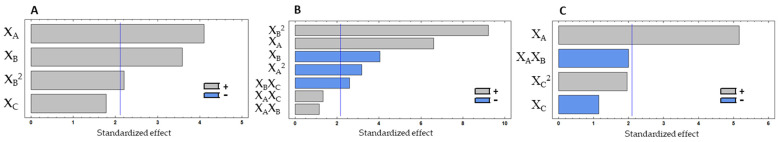
Pareto charts with the effect of three experimental factors, in decreasing order, obtained for protein % (**A**), antioxidant (**B**) and anti-hypertensive (**C**) properties in the experimental design with corolase, showing the most influent factors. The vertical lines in the pareto charts represent the level of significance (*p* = 0.05). X_A_—Enzyme %; X_B_—Hydrolysis temperature; X_C_—Hydrolysis time.

**Figure 4:**
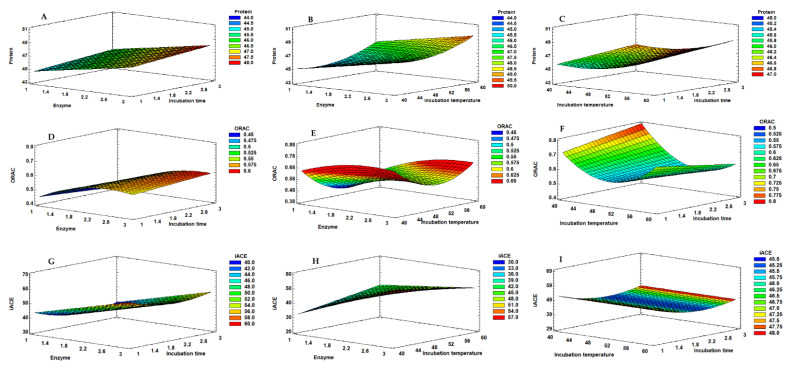
Response surface graphs corresponding to the combined effect of enzyme % (XA), hydrolysis temperature (XB) and hydrolysis time (XC), on the protein (**A**–**C**), ORAC (**D**–**F**) and iACE (**G**–**I**) responses obtained for the mussel extracts produced with corolase.

The experimental design matrix and the responses obtained for the Box–Behnken factorial design performed with the corolase protease are shown in Table 7. Figure 3 shows the Pareto charts, and Figure 4 shows the response surface graphics obtained for the independent variables tested, showing their interactions when studying each dependent variable.

The protein content of the hydrolysates showed a variation in the range of 44.30–49.77%. By analysing the Pareto chart (Figure 3A), protein% was positively influenced by the linear effect of enzyme% (X_A_) and temperature (X_B_), meaning that the increase in enzyme concentration and temperature leads to an increase of protein release from the mussel biomass. The time of the hydrolysis did not significantly affect the protein % (*p* > 0.050). The quadratic effect of the temperature was also positively significant (*p* < 0.050). By multiple regression, the predicted response for the protein % could be obtained by the model in Equation (4).

Protein % = 61.4391 + 1.20687 × X_A_ − 0.846161 × X_B_ + 0.00951786 × X_B_^2^ (4)

The final adjusted model showed a significant fit (*p* > 0.050) (Table 8). Still, the R^2^ indicates that only about 60% of the variability observed in relation to the protein content is explained by the model, indicating that the increase of corolase %, temperature, and hydrolysis time is not beneficial to produce protein-rich hydrolysates.

The antioxidant activity was significantly influenced by the linear effect of enzyme% (X_A_) and temperature (X_B_) (*p* < 0.050), while the hydrolysis time (X_C_) did not show a significant effect (*p* > 0.050). Enzyme% showed a positive effect, meaning that the increase in enzyme concentration results in higher ORAC values. On the other hand, temperature had a negative effect. Thus, the higher the temperature the lower the ORAC values will be. Furthermore, the quadratic effect of both factors also showed a significant effect. The linear interaction between temperature and hydrolysis time had a significant negative effect (Figure 3B).

By multiple regression, the predicted response for the ORAC could be obtained by the model in Equation (5).

ORAC = 4.11383 + 0,158065 × X_A_ − 0.153981 × X_B_ − 0.0548188 × X_A_^2^ + 0.00154318 × X_B_^2^ − 0.0045× X_B_ × X_C_ (5)

The final adjusted model highly explains the antioxidant activity, showing a significant fit (*p* > 0.050) and R^2^ = 87.09% (Table 9).

The ACE inhibition (iACE) % was evaluated in a concentration of 10 mg hydrolysate/mL. The only variable that showed a significant effect on iACE was the enzyme% (X_A_), with a positive effect (Figure 3C). Thus, the increase in enzyme concentration increases the anti-hypertensive potential of the hydrolysates, which may be explained by the formation of more peptides with the ability to inhibit the ACE.

By multiple regression, the predicted response for the iACE could be obtained by the model in Equation (6).

iACE = 4.11383 + 0.158065 × X_A_ (6)

The ANOVA results for the adjusted model was verified to have a significant fit (*p* > 0.050) (Table 10). However, the model only explained 57.90% of the variability in the anti-hypertensive results.

The Box–Behnken design allowed to optimise the conditions that would enable higher results for the individual responses (Table 11). However, a Derringer’s desirability analysis was performed to optimise multiple responses of the design (Table 11). Thus, the hydrolysis of the minced mussel meat with 3% of the enzyme, at 40 °C for 3 h, seems to represent the best conditions to obtain the higher results in terms of hydrolysate proteins/peptides content as well as antioxidant and anti-hypertensive properties.

An enzymatic hydrolysis was performed, in triplicate, using the optimised conditions according to the design for the purpose of validation. The experiment was performed using the exact quantities used in the design experiments. The temperature was adjusted to 40 °C to work practically. The obtained hydrolysates were freeze-dried and then evaluated regarding their protein content and antioxidant and anti-hypertensive potential. The hydrolysates showed a mean of 47.36 ± 1.02% of protein content, antioxidant activity of 0.65 ± 0.062 µmol TE/mg hydrolysate, and ability to inhibit the activity of ACE in 55.36 ± 2.12% (at 10 mg hydrolysate/mL). The obtained results, although slightly lower, were not so different from the predicted ones. Furthermore, a scale-up hydrolysis was performed, in triplicate, with an increase of 15 times the amount of mussel biomass and solvent, maintaining the ratio used in the experimental design. The final hydrolysates showed a mean of 46.35 ± 1.12% of protein content, antioxidant activity of 0.389 ± 0.021, and IC_50_ for ACE inhibition of 3.7 ± 0.33 mg protein/mL (Table 12). The scaled-up results were verified to be slightly lower than the predicted ones and the validation hydrolysates regarding protein content. However, the antioxidant activity showed a pronounced decrease.

## 3. Discussion

Mussel meat has a high protein content, making it interesting to produce bioactive hydrolysates rich in proteins and bioactive peptides. However, the mussel *Mytilus galloprovincialis* is less exploited regarding its bioactive potential when compared to other mussel species, such as *M. coruscus* and *M. edulis*. Since we wanted to create a food-grade method, we chose two food-grade proteases to carry out enzymatic hydrolysis. Thus, to explore this mussel potential, we have performed two Box–Behken experimental designs, with two different proteases, aiming to obtain hydrolysates with interesting potential for industrial applications. Furthermore, we have not found studies with mussels from the genus *Mytilus* performing enzymatic hydrolysis with subtilisin or corolase. The most frequent enzymes found were mainly gastric enzymes, such as pepsin and trypsin, and non-gastric enzymes, such as papain and flavourzyme.

The mussel meat biomass used showed 70.50 ± 13.44% of protein on a dry weight (DW) basis and a moisture content of 90.30 ± 4.24%. These results show higher values of protein when compared to other studies with *Mytilus* sp. from Portugal and Spain that showed protein content variation from 39.17–42.94 (DW) and moisture % of 81.71–87.59% [23]. However, these results are in line with the possible variations in protein content that can occur in different months, as shown by Çelik [24] in a study with *Mytilus galloprovincialis* indicating higher protein levels (74.64%) in February.

The protein % of the hydrolysates does not seem to be highly influenced by the determined models, indicating that enzymatic hydrolysis with both enzymes can produce hydrolysates with protein contents in the range of 40–48% (DW). So, to obtain mussel hydrolysates with a content of above 40%, the most economical and fastest conditions can probably be used.

The subtilisin protease optimised method was an enzymatic hydrolysis with 1.5% of enzyme with a duration of 3 h at 52 °C. In a scale-up test with these conditions, the final hydrolysates showed protein content, ORAC, and IC50 for ACE inhibition values of 45.0 ± 0.38, 0.49 ± 0.061 245 μmol TE/mg hydrolysate, and 1.0 ± 0.56 mg protein/mL, respectively. With the corolase, the optimised method was an enzymatic hydrolysis with 3.0% of enzyme with a duration of 3 h at 40 °C, obtaining scale-up hydrolysates with protein content, ORAC, and IC_50_ for ACE inhibition values of 46.35 ± 1.12, 0.389 ± 0.021 μmol TE/mg hydrolysate, and 3.7 ± 0.33 mg protein/mL, respectively. The experimental design responses were not highly explained by the models, indicating that the system is highly variable, as necessary to enhance the process, or a plateau may have been quickly reached, which challenges the explanation of the variability in the models. However, the hydrolysates showed potential as proteins/peptides sources with antioxidant properties, bringing interest to the results. In both experiments, interesting protein values were obtained with a few hours of hydrolysis, which is in line with other studies showing that enzymatic hydrolysis with papain for 2 h was enough for achieving the maximum protein extraction [25]. The obtained protein content (450 and 463 mg protein/g hydrolysate) was close to those obtained for *Mytilus edulis* by Vareltzis and Undeland (430 and 580 mg protein/g with acid and alkaline process, respectively) [26], but lower than those obtained by Neves et al. (735.45 ± 11.45 mg protein/g hydrolysate) [15]. The subtilisin method needs a lower enzyme% to obtain higher bioactive properties than the corolase, with the main difference being observed for the anti-hypertensive potential. Even though the corolase hydrolysate shows a higher protein %, this does not bring much potential for this hydrolysate due to the small difference compared to the subtilisin hydrolysate. So, mussel meat hydrolysate produced with the subtilisin protease appears to have more potential for further studies as an active ingredient, at least regarding the antioxidant and anti-hypertensive potential. However, it is important to highlight that the obtained values for the anti-hypertensive property are not very significant since IC_50_ ≥ 1000 µg protein/mL [27]. The hydrolysate produced with corolase shows the lowest potential with an IC_50_ = 3700 µg protein/mL. The subtilisin hydrolysate seems to be more promising, with an IC_50_ = 1000 µg protein/mL. Bioactive peptides usually have a molecular weight (MW) less than 6 KDa [28], and the most efficient anti-hypertensive peptides are usually associated with MW lower than 3 KDa [29]. Several marine derived peptides with MW lower than 3 KDa have been described, such as the microalgae *Chlorella vulgaris* VECYGPNRPQF peptide (1.3 KDa; IC_50_ of 29.6 µM) [30] and *C. ellipsoidea* VEGY peptide (467 Da; IC_50_ of 128.4 µM) [31]; the macroalgae *Gracilariopsis lemaneiformis* TGAPCR peptide (604 Da; IC_50_ of 23.94 µM) [32] and *Nannochloropsis oculata* LEQ peptide (369 Da; IC_50_ of 173 µM) [33]. Thus, to increase the anti-hypertensive potential of the produced hydrolysates, a future approach may be to submit them to a ultrafiltration system using 3-KDa cut-offs, to concentrate peptides with lower MW [34]. Furthermore, the production of low MW peptides may also increase the antimicrobial potential of hydrolysates, thus presenting new possible applications.

All the hydrolysis performed used the same mussel batch, initially minced and stored at −20 °C. The main goal was to assure that all the hydrolysis were performed with minimum mussel internal variations, since we wanted to compare a large number of extracts to optimize the hydrolysate production. The validation and scaled-up hydrolysis were also performed with the same batch, allowing us to precisely compare these extracts with those obtained using the experimental design, excluding possible mussel internal chemical variations. However, it is important to point out that mussel meat biochemical composition varies with the harvesting season, due to their reproductive cycle, environmental conditions, growth, and food availability [24]. Çelik et al. [24] showed that mussel protein content is highly related with the spawning seasons, with decreased protein levels being observed during this season, which increases after spawning time. So, different harvesting seasons lead to variations in the biochemical composition, which may be reflected in differences in mussel protein and amino acids, not only in terms of quantity, but also quality. Consequently, the enzyme action will produce different peptides over the seasons. Therefore, it would be expected that the ORAC and iACE results obtained for hydrolysates produced with the presented methods may differ between different mussel batches, depending on their harvesting season and other external factors. Furthermore, the mussel’s digestive gland produces proteases, which also seems to be influenced by their diet [35], and mussels seem able to modulate their digestive enzyme activities in response to limited feeding and thermal stress [36]. Since endogenous proteases may also have either a proteolytic effect or serve as an enzymatic substrate in the hydrolysis, the amount of endogenous proteases may also contribute to the variability of results. Thus, in the future, it would be of great interest to perform the same hydrolysis in different mussel batches, harvested in different months, and perhaps from different locations, to examine the variability of the produced hydrolysates when influenced by the expected biochemical composition differences.

The production of multifunctional extracts from mussels may be an interesting approach for food applications since they are not only a source of proteins, but also present bioactivities that can enhance consumer health, useful for the creation of functional food. Moreover, they may also be used as nutraceuticals or as cosmetic ingredients. Antioxidant food and nutraceuticals may help reduce levels of radical oxygen species that are constantly produced by the human organism, especially during high exposure to external factors, such as alcohol, tobacco smoke, and stress [37]. Hypertension has been associated as one of the main causes of cardiovascular diseases [38], with the angiotensin-converting enzyme (ACE) being one of the major enzymes involved in the process of blood pressure regulation [39]. Thus, multifunctional extracts may be incorporated in food matrices with health claims, to facilitate sale as functional food. However, for claiming health benefits, it is important to study the bioavailability of food matrices incorporating these hydrolysates [40], by analysing their resistance to the gastrointestinal (GI) tract enzymes and conditions, to verify if their properties are maintained throughout the GI tract passage [41]. In cosmetics, antioxidants are especially important for anti-ageing purposes since free radicals are highly associated with skin ageing. Thus, natural antioxidant hydrolysates used as active cosmetic ingredients may help decrease free radical damage and work as an alternative for synthetic antioxidant ingredients.

Furthermore, mussel protein and peptide hydrolysates are frequently associated with other properties, especially antimicrobial properties [42,43], but also anticancer [44], anti-inflammatory [45], anticoagulant [14], antidiabetic [15], and antiviral [46]. Thus, in the future, it would be interesting to study these hydrolysates for other bioactivities. Additionally, the water-soluble nature of these extracts makes it easy to incorporate them in several matrices. Although the freeze-drying process may lead to a loss of bioactivity, it is important for a better preservation of the hydrolysates, facilitating their incorporation in both solid and liquid matrices.

## 4. Materials and Methods

### 4.1. Materials

The enzymes used were subtilisin kindly supplied by Aquitex, and the commercial digestive-enzyme complex Corolase PP purchased from AB Enzymes GmbH (Darmstadt, Germany). The mussels were kindly supplied by Testa & Cunhas (Gafanha da Nazaré, Portugal).

### 4.2. Mytilus Galloprovincialis Meat Characterisation

The *Mytilus galloprovincialis* meat used was characterised, in triplicate, before being minced, according to a few nutritional characteristics. Total fat, protein, and moisture content were measured in accordance with the established standards PE.Q.AC.04 Ed.06, PE.Q.AC.03 Ed.07 (ISO 1871:2009), and PE.Q.AC.01 Ed.06 (NP 2282:2009), respectively.

### 4.3. Enzymatic Hydrolysis Procedures

When received, mussels were clean, and the meat was separated from the shell. Mussel meat was then minced until homogenised and stored at −20 °C for further analysis. A preliminary study was performed with different conditions, with variations on the enzymes concentration (0.5–4%), hydrolysis time (from 30 min to 4 h), and mussel/water ratio (*w*:*v*) (1:1, 1:2, 1:3), to understand the limits to be established for the experimental design. Concerning the experimental design, all the hydrolysis reactions for both enzymes were prepared using the previously stored mussel meat minced biomass. Briefly, mussel biomass was mixed with ultrapure water in a ratio of mussel:water of 1:2 (*w*:*v*) and pH was adjusted to 7.5. Then, the enzyme was added in the intended test concentration and the mixtures were incubated at the test temperature in an orbital shaker (Thermo Scientific™ MaxQ™ 6000) (conditions tested at Table 1 and Table 7). The pH was verified and adjusted to 7.5 every 15 min. To stop the hydrolysis reaction, the samples were incubated at 90 °C for 10 min to inactivate the enzymes. Samples were centrifuged at 5000× *g* for 30 min, and the supernatant was collected and freeze-dried for further analysis.

### 4.4. Experimental Design

Two experimental designs, one with corolase and the other with the subtilisin protease, were implemented to establish the most influential factors that could produce a hydrolysate rich in proteins and bioactive properties. For that, a Box–Behnken design was selected. The factors evaluated were enzyme %, hydrolysis temperature (°C), and hydrolysis time (hours). Enzyme % and hydrolysis time were chosen according to single-factor experiments (data not shown). The ORAC assay was performed for each hydrolysate. The temperature and the pH tested were selected according to the functioning range of the enzymes. The levels of the factors coded as −1, 0, and 1 were established and are shown in Table 13. The selected response variables were protein content as well as antioxidant and anti-hypertensive potential. Each design resulted in an arrangement of 15 treatments, executed in duplicate (a total of 30 runs). Each hydrolysis was performed as described before.

### 4.5. Statistical Analysis and Statistical Model

The optimisation analysis was performed using Statgraphic Centurion software. All data were expressed as means ± standard deviation (S.D.). Means were considered statistically significant using a significance level of 0.05. Responses were adjusted to the second-order polynomial model (Equation (7)):

Y = β_0_ + β_A_X_A_ + β_B_X_B_ + β_C_X_C_ + β_A,B_X_A_X_B_ + β_A,C_X_A_X_C_ + β_B,C_X_B_X_C_ + β_A,A_X_A_^2^ + β_B,B_X_B_^2^ + β_C,C_X_C_^2^ + ε (7)

where Y is the measured response; β0 is the constant; βA–βC are the coefficients associated with linear, quadratic, and interaction effects of the variables X_A_ (enzyme %)_,_ X_B_ (Temperature), and X_C_ (Time), respectively, and ε is the residual error. In the final models for each variable, only the significant effects appear (*p* < 0.05). To optimise the multiple responses obtained, a Derringer’s desirability function was applied to the results of each design [22].

### 4.6. Protein Quantification

Total nitrogen content was determined by the micro-Kjeldahl method. Briefly, 0.2 g of freeze-dried hydrolysate were digested with 1 g of Kjeldahl catalyst and 4 mL of H_2_SO_4_ (ρ20 = 1.84 g/mL) at 400 °C for 2 h. The reaction was stopped with 20 mL of deionised water. The samples were distilled using 30 mL of NaOH 10 M. A boric acid solution with bromocresol and methyl red was used as indicator. The resulting solution was titrated with HCl 0.1 M. The total nitrogen and protein percentage were determined using the Equations (8) and (9), respectively, where f (HCl 0.1 m) = 0.0014 and Kjeldahl factor = 6.25.

Total nitrogen (%) = f × (V_sample_ − V_blank_) × (100/sample weight) (8)

Protein content (%) = Total nitrogen (%) × 6.25(9)

### 4.7. Antioxidant Activity

The antioxidant activity was measured by the Oxygen radical absorbance capacity (ORAC) assay, performed in a black 96-well microplate (Nunc, Denmark) according to the method described by Coscueta et al. (2020) [47]. Briefly, the reaction was carried out in 75 mM phosphate buffer (pH 7.4) at 40 °C. The final assay mixture was 200 µL, containing fluorescein (70 nM, final concentration in well), 2′-Azobis (2-methylpropionamidine) dihydrochloride (AAPH) (12 mM, final concentration in well), and either Trolox (1–8 µM, final concentration in well), for the calibration curve, or sample. A control with PBS instead of the antioxidant solution was used. Before adding AAPH, the mixture was pre-incubated for 10 min at 37 °C. AAPH solution was added rapidly. The fluorescence was recorded at intervals of 1min for 80 min in a multidetection plate reader (Synergy H1; BioTek, Winooski VT, USA) with excitation and emission wavelengths of 485 nm and 528 nm, respectively. The equipment was controlled by the Gen5 BioTek software version 3.04. Antioxidant curves (fluorescence versus time) were first normalised to the curve of the blank corresponding to the same assay by multiplying original data by the factor fluorescence blank, t = 0/fluorescence control, t = 0. The area under the fluorescence decay curve (AUC) was calculated according to the trapezoidal method from the normalised curves. The final AUC values were calculated by subtracting the AUC of the blank from all the results. Regression equations between net AUC and antioxidant concentration were calculated.

### 4.8. Anti-Hypertensive Activity

The ACE-inhibitory activity was performed in a black 96-well microplate (Nunc, Denmark) according to the method described by Sentandreu & Toldrá (2006) [48] with some modifications [27]. This method is based on the ability of the angiotensin-I converting enzyme (ACE) to hydrolyse a specific substrate (o-aminobenzoylglycyl-p-nitrophenylalanylproline (Abz–Gly–Phe(NO2)–Pro)), generating the fluorescent product o-aminobenzoylglycine (Abz–Gly). A commercial Angiotensin-I converting enzyme (EC 3.4.15.1, 5.1 U/mg), purchased from Sigma Chemical (St. Louis, MO, USA), was previously diluted in 5 mL of a glycerol solution in 50% ultra-pure water. Then, ACE was diluted 1:24 with a 150 mM Tris buffer solution (pH 8.3), containing 1 µM of ZnCL2, reaching a final concentration of 42 mU/mL). A total of 40 µL of ultrapure water or ACE working solution was added to each microtiter-plate well, then adjusted to 80 µL by adding ultrapure water to blank, control, or samples. The reaction was initiated with the addition of 160 µL of the substrate solution (0.45 mM solution of ABz-Gly-Phe(NO2)-Pro (Bachem Feinchemikalien, Bubendorf, Switzerland) dissolved in 150 mM Tris buffer (pH 8.3) containing 1.125 M NaCl). The mixture was incubated at 37 °C for 30 min, and the fluorescence generated was measured using a multidetection plate reader (Synergy H1; BioTek, Winooski VT Vermont, USA) with excitation and emission wavelengths of 350 nm and 420 nm, respectively. To obtain the IC_50_ of the inhibitory activity, which is the concentration of the sample that is required to inhibit the original ACE activity by 50%, serial dilutions of each sample were performed (1/1 to 1/32). A non-linear modelling of the obtained data was used to calculate the IC_50_ values, using the 5 Parameter curve fit method and the Interpolate function from Gen5 software (BioTek Instruments).

## 5. Conclusions

Although marine species have often been described as a source of bioactive hydrolysates and bioactive peptides, the mussel *Mytilus galloprovincialis* has been less exploited. Due to its high protein level, this marine specie seems to be an interesting potential source of bioactive peptides. Thus, in this work, the factorial designs allowed to confirm the combination of experimental factors that leads to the production of the most efficient hydrolysate from the mussel *Mytilus galloprovincialis,* with the highest levels of proteins/peptides as well as antioxidant and anti-hypertensive activity. The use of enzymatic hydrolysis with food-grade enzymes presents the opportunity to create active ingredients that can be further explored to produce functional food, nutraceuticals, and cosmetics. Furthermore, the use of discarded mussels to produce functional ingredients for food, cosmetic, and pharmaceutic industries may contribute to the valorisation of world waste in a circular economy context.

## Figures and Tables

**Table 1:** Box-Behnken factorial design matrix for three factors and three responses obtained for the subtilisin protease.

Run	Factors			Response ^1^		
	% Enzyme	Hydrolysis Temperature (°C)	Hydrolysis Time (h)	Protein Content (%)	ORAC (µmol TE/mg)	ACE Inhibition (%)
1	0.5	40	2	45.56 ± 0.38	0.49 ± 0.08	49.89 ± 18.86
2	0.5	50	3	49.11 ± 0.41	0.60 ± 0.05	56.21 ± 2.39
3	1.5	40	2	47.17 ± 0.98	0.55 ± 0.06	44.51 ± 17.37
4	1.5	60	2	45.87 ± 2.79	0.72 ± 0.07	65.55 ± 1.77
5	0.5	50	1	48.83 ± 0.93	0.59 ± 0.02	63.86 ± 0.07
6	1	50	2	48.97 ± 1.86	0.60 ± 0.04	59.97 ± 3.64
7	1.5	50	3	48.03 ± 2.18	0.60 ± 0.05	72.74 ± 2.24
8	1	60	3	47.66 ± 2.21	0.66 ± 0.00	66.30 ± 0.41
9	1	40	1	45.04 ± 2.80	0.54 ± 0.02	61.31 ± 0.57
10	1	40	3	46.68 ± 2.71	0.56 ± 0.04	47.59 ± 3.73
11	1	60	1	45.66 ± 2.94	0.61 ± 0.07	69.48 ± 0.85
12	1.5	50	1	46.86 ± 1.25	0.59 ± 0.01	67.67 ± 0.48
13	0.5	60	2	48.80 ± 0.60	0.56 ± 0.04	67.62 ± 3.53
14	1	50	2	49.02 ± 0.02	0.64 ± 0.06	51.62 ± 2.04
15	1	50	2	49.12 ± 0.14	0.62 ± 0.04	60.41 ± 1.65

^1^ Values expressed as mean ± SD of two replicates.

**Table 2:** Analysis of variance (ANOVA) for protein % obtained for the subtilisin Box-Behken design.

Model	Sum of Squares	DF	Mean Square	F Value	*p*-Value
X_A_ (Enzyme %)	4.7524	1	4.7524	1.80	0.1955
X_C_ (Time)	6.4516	1	6.4516	2.44	0.1345
X_A_X_B_	10.2378	1	10.2378	3.88	0.0637
X_B_^2^	30.0804	1	30.0804	11.39	0.0032
Lack of fit	7.94221	6	1.3237	0.50	0.7995
Pure error	50.16	19	2.64		
Total	109.624	29			
R^2^ = 47.00, Adj − R^2^ = 38.52, CV = 1.62					

**Table 3:** Analysis of variance (ANOVA) for ORAC results obtained for the subtilisin Box-Behken design.

Model	Sum of Squares	DF	Mean Square	F Value	*p*-Value
X_A_ (Enzyme %)	0.0120506	1	0.0120506	6.20	0.0212
X_B_ (Temperature)	0.0422508	1	0.0422508	21.75	0.0001
Lack of fit	0.0124529	3	0.00415097	2.14	0.1259
Pure error	0.0407955	21	0.00194264		
Total	0.118375	29			
R^2^ = 55.02, Adj − R^2^ = 45.65, CV = 0.044					

**Table 4:** Analysis of variance (ANOVA) for ACE inhibition (%) obtained for the subtilisin Box-Behken design.

Model	Sum of Squares	DF	Mean Square	F Value	*p*-Value
X_A_ (Enzyme %)	315.868	1	315.868	29.62	0.0001
X_B_ (Temperature)	855.895	1	855.895	80.27	0.0000
X_C_ (Time)	94.9163	1	94.9163	8.90	0.0093
X_A_ X_B_	184.73	1	184.73	17.32	0.0008
X_A_ X_C_	81.0901	1	81.0901	7.60	0.0147
X_B_ X_C_	55.5458	1	55.5458	5.21	0.0375
X_C_^2^	230.418	1	230.418	21.61	0.0003
Lack of fit	85.5525	3	28.5175	2.67	0.0848
Pure error	159.948	15	10.6632		
Total	1969.71	27			
R^2^ =87.54, Adj − R^2^ = 81.30, CV = 3.26					

**Table 5:** Optimal conditions predicted by the experimental design to maximise protein/peptide release and antioxidant and anti-hypertensive activities of the hydrolysates, and Derringer desirability to predict the optimal conditions for a multiple response.

Factors	Response			
	Protein	ORAC	iACE	Erringer Desirability
Temperature (°C)	52.8	60.0	59.9	52.5
Protease (%)	0.5	1.5	1.5	1.5
Hydrolysis time (h)	3.0	2.0	3.0	3.0

**Table 6:** Results predicted by the model, and results obtained in a validation and a scaled-up enzymatic hydrolysis, performed with the optimal conditions described in Table 5.

Evaluated Characteristics	Predicted Results	Obtained Results	
		Validation	Scaled-Up
Protein (%)	48.22	46.70 ± 0.36	45.0 ± 0.38
Antioxidant activity (ORAC) (µmol TE/mg hydrolysate)	0.64	0.54 ± 0.029	0.49 ± 0.061
Anti-hypertensive activity (% inhibition at 2.5 mg/mL)	71.87	70.21 ± 2.9	_____
Anti-hypertensive activity (IC_50_ mg of protein/mL)	_____	_____	1.0 ± 0.56

**Table 7:** Box-Behnken factorial design matrix for three factors and three responses obtained for the corolase protease.

Run	Factors			Response ^1^		
	% Enzyme	Hydrolysis Temperature (°C)	Hydrolysis Time (h)	Protein Content (%)	ORAC (µmol TE/mg)	ACE Inhibition (%)
1	3	40	2	47.27 ± 0.21	0.76 ± 0.04	61.10 ± 5.43
2	2	50	3	47.38 ± 1.37	0.46 ± 0.06	46.79 ± 7.94
3	2	40	2	45.84 ± 0.28	0.57 ± 0.02	46.13 ± 6.34
4	2	60	2	48.33 ± 0.65	0.59 ± 0.01	48.44 ± 2.09
5	3	50	1	47.39 ± 0.02	0.55 ± 0.00	59.40 ± 1.36
6	2	50	2	45.56 ± 2.40	0.57 ± 0.01	43.43 ± 9.55
7	3	50	3	49.77 ± 0.49	0.60 ± 0.17	49.34 ± 17.73
8	1	60	3	47.26 ± 2.29	0.52 ± 0.02	38.80 ± 4.13
9	1	40	1	46.42 ± 1.28	0.42 ± 0.06	40.40 ± 8.85
10	1	40	3	44.64 ± 0.99	0.63 ± 0.11	32.19 ± 3.76
11	2	60	1	47.38 ± 1.09	0.67 ± 0.16	49.66 ± 7.55
12	2	50	1	45.85 ± 0.74	0.67 ± 0.15	50.21 ± 4.48
13	2	60	2	48.23 ± 0.91	0.72 ± 0.00	52.33 ± 4.90
14	1	50	2	44.30 ± 0.41	0.46 ± 0.04	45.72 ± 4.09
15	3	50	2	47.85 ± 0.85	0.59 ± 0.02	54.23 ± 3.00

^1^ Values expressed as mean ± SD of two replicates.

**Table 8:** Analysis of variance (ANOVA) for protein % obtained for the corolase Box-Behken design.

Model	Sum of Squares	DF	Mean Square	F Value	*p*-Value
X_A_ (enzyme %)	23.3048	1	23.3048	16.79	0.0008
X_B_ (Temperature)	17.8506	1	17.8506	12.86	0.0023
X_B_^2^	6.76402	1	6.76402	4.87	0.0413
Lack of fit	3.91492	8	0.489365	0.35	0.9315
Pure error	23.5968	17	1.38805		
Total	79.8516	29			
R^2^ = 65.54, Adj − R^2^ = 60.03, CV = 1.17					

**Table 9:** Analysis of variance (ANOVA) for ORAC obtained for the corolase Box-Behken design.

Model	Sum of Squares	DF	Mean Square	F Value	*p*-Value
X_A_ (enzyme%)	0.0781744	1	0.0781744	43.67	0.0000
X_B_ (Temperature)	0.0293709	1	0.0293709	16.41	0.0014
X_A_^2^	0.0181707	1	0.0181707	10.15	0.0072
X_B_^2^	0.152151	1	0.152151	85.00	0.0000
X_B_ X_C_	0.01215	1	0.01215	6.79	0.0218
Lack of fit	0.00655055	5	0.00131011	0.73	0.6122
Pure error	0.02327	13	0.00179		
Total	0.320938	25			
R^2^ = 90.71, Adj − R^2^ = 87.09, CV = 0.04					

**Table 10:** Analysis of variance (ANOVA) for ACE inhibition (%) obtained for the corolase Box-Behken design.

Model	Sum of Squares	DF	Mean Square	F Value	*p*-Value
X_A_ (enzyme %)	1120.74	1	1120.74	26.62	0.0001
Lack of fit	58.3703	6	9.72839	0.23	0.9612
Pure error	799.952	19	42.1027		
Total	2364.9	29			
R^2^ = 63.71, Adj − R^2^ = 57.90, CV = 6.49					

**Table 11:** Optimal conditions predicted by the experimental design to maximise protein/peptide release and antioxidant and anti-hypertensive activities of the hydrolysates, and Derringer desirability to predict the optimal conditions for a multiple response.

Factors	Response			
	Protein	ORAC	iACE	Derringer Desirability
Temperature (°C)	60.0	40.0	40.0	40.1
Protease (%)	3.0	2.7	3.0	3.0
Hydrolysis time (h)	3.0	3.0	1.0	3.0

**Table 12:** Results predicted by the model, and results obtained in a validation and scaled-up enzymatic hydrolysis, performed with the optimal conditions described in Table 11.

Evaluated Characteristics	Predicted Results	Obtained Results	
		Validation	Scaled-Up
**Protein (%)**	48.01	47.36 ± 1.06	46.35 ± 1.12
Antioxidant activity (ORAC) (µmol TE/mg hydrolysate)	0.82	0.65 ± 0.062	0.389 ± 0.021
Anti-hypertensive activity (% inhibition at 5 mg/mL)	61.10	55.36 ± 2.12	_____
Anti-hypertensive activity (IC_50_ mg of protein/mL)	_____	_____	3.7 ± 0.33

**Table 13:** Levels for 3 experimental factors for the two experimental designs.

Factors	Subtilisin				Corolase	
	−1	0	1	−1	0	1
% Enzyme (X_A_)	0.5	1	1.5	1	2	3
Hydrolysis temperature (°C) (X_B_)	40	50	60	40	50	60
Hydrolysis time (h) (X_C_)	1	2	3	1	2	3

## Data Availability

Not applicable.

## Sample Availability

Samples of the compounds are not available from the authors.
